# The infection characteristics and autophagy defect of dermal macrophages in STZ‐induced diabetic rats skin wound *Staphylococcus aureus* infection model

**DOI:** 10.1002/iid3.492

**Published:** 2021-10-14

**Authors:** Xiaoying Xie, Rihui Zhong, Ling Luo, Xianghua Lin, Lisi Huang, Songyin Huang, Lijia Ni, Baiji Chen, Rui Shen, Li Yan, Chaohui Duan

**Affiliations:** ^1^ Department of Clinical Laboratory, Sun Yat‐Sen Memorial Hospital Sun Yat‐Sen University Guangzhou Guangdong China; ^2^ Guangdong Provincial Key Laboratory of Malignant Tumor Epigenetics and Gene Regulation, Sun Yat‐Sen Memorial Hospital Sun Yat‐Sen University Guangzhou Guangdong China; ^3^ Department of Endocrinology, Sun Yat‐Sen Memorial Hospital Sun Yat‐Sen University Guangzhou Guangdong China

**Keywords:** autophagy, diabetic foot infection, intracellular infection, *Staphylococcus aureus*

## Abstract

**Introduction:**

Diabetic foot ulcer infection (DFI) is an infectious disease of the skin and soft tissue in diabetics notorious for making rapid progress and being hard to cure. *Staphylococcus aureus* (*S. aureus*), most frequently detected in DFI, recently was suggested as an intracellular pathogen that can invade and survive within mammalian host cells. Autophagy in macrophages plays a vital immune role in combating intracellular pathogens through bacterial destruction, but there is a lack of empirical research about the infection characteristics and autophagy in diabetic skin infection.

**Methods:**

Here, we used streptozotocin‐induced Sprague Dawley rats as a diabetic skin wound model to examine the *S. aureus* clearance ability and wound healing in vitro. Western blot and immunofluorescence staining were used to evaluate the autophagic flux of the macrophages in diabetic rats dermis, even with *S. aureus* infection.

**Results:**

We demonstrated that infections in diabetic rats appeared more severe and more invasive with weakened pathogen clearance ability of the host immune system, which coincided with the suppressed autophagic flux in dermal macrophages, featured by a significant increase in endogenous LC3II/I and in p62.

**Conclusions:**

Our results first provided convincing evidence that autophagy of macrophages was dysfunctional in diabetes, especially after being infected by *S. aureus*, which weakens the intracellular killing of *S. aureus*, potentially worsens the infections, and accelerates the infection spread in the diabetic rat model. Further understanding of the special immune crosstalk between diabetes host and *S. aureus* infection through autophagic factors will help to explain the complex clinical phenomenon and guarantee the development of effective therapies for diabetic foot infections.

## Graphical abstract

Infections in diabetic rats appeared more severe and more invasive with weakened pathogen clearance ability of the host immune system, which coincided with the suppressed autophagic flux in dermal macrophages, featured by a significant increase in endogenous LC3II/I and in p62. Our results provided convincing evidence that autophagy of macrophages was dysfunctional in diabetes, especially after being infected by *Staphylococcus aureus* (*S. aureus*), which weakens the intracellular killing of *S. aureus*, potentially worsen the infections, and accelerates the infection spread in the diabetic rat model.

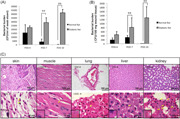

## 1 INTRODUCTION

Foot ulcers are very common in diabetic patients with a high prevalence between 15% and 25%.^1^ The ulcer infections are frequent and were a leading cause of death and crippling.^2^ Diabetic foot infection (DFI) is estimated to be the most common reason for lower‐limb amputations and causes a large social and economical burden.^3^, ^4^, ^5^

*Staphylococcus aureus* (*S. aureus*) is the pathogen most frequently isolated in DFI,^6^ with high drug resistance, severe invasiveness, and poor prognosis. It was regarded as an intracellular pathogen recently,^7^ and could escape from the phagolysosomal pathway into the cytoplasm. This strategy allows the extracellular bacteria to become intracellular bacteria, and then replicate and survive with the consequent killing of the eukaryotic host cell and spreading of the infection,^8^, ^9^ which is called “bacterial internalization.” Different from the extracellular bacteria, intracellular bacteria can escape routine antibiotics under the “protection” of host cells, additionally can disseminate to the deeper tissue and distant organs with the migration of host cells. Bacterial internalization brought a series of problems to clinical infection treatment, including antibiotics being invalid, rapid deteriorating infection, and chronic infection, prominently in DFI patients. Therefore, to investigate the infection, the study of the characteristics and clearance of *S. aureus* in DFI was necessary.

As the first physical and immunological defense line, the skin is the body's most exposed environmental interface. Recently, a new contribution in immune response for several populations of cells residing in different layers of the skin has emerged. The immune system within the skin is located in both major structural compartments, the epidermis and dermis, and consists of several important types of immune‐competent cells.^10^ Macrophages are a major subpopulation of cells in the dermis, and what is important, the first defense line against pathogens.^11^, ^12^ When a pathogen enters the broken skin and causes infection, the macrophages can migrate to the infection site to clear the pathogens via xenophagy and phagocytosis.

Xenophagy plays an essential role in host defense against invading pathogens,^13^, ^14^, ^15^ also known as antibacterial autophagy. Autophagy is an intracellular process that involves the degradation and recycling of defective cytosolic proteins. Besides this housekeeping function, the ability of autophagy to selectively target intracellular pathogens for destruction is now regarded as a key aspect of the innate immune response. Accordingly, the autophagy of professional phagocytes, as macrophages, plays a vital role in combating intracellular pathogens.^16^ Therefore, the knowledge of autophagy change against intracellular pathogens in the macrophages in diabetes is important to reveal the causes of incurable DFI, while remaining poorly elucidated.

Our in vitro study found that advanced glycation end products (AGEs), a set of adducts formed on proteins by glycation with reducing sugars such as glucose in diabetics, impaired autophagic flux of the host macrophages by inducing autophagosome formation but blocking the autophagosome‐lysosome fusion, which diminished intracellular bactericidal capability of macrophages, and is likely to increase the invasiveness of *S. aureus* in DFI,^17^ but this has not been verified in vivo. In this study, we test the wound bacterial burden, the invasive and disseminate capacity of *S. aureus* in diabetic rat skin infection to investigate DFU infection characteristics, and examined the autophagic flux in diabetic rats' infected wound skin and dermis macrophages, tried to investigate the potential relationship between the autophagy and the unique infection characteristic in diabetics. And we showed that the diabetic rats appeared to have similar features as the diabetic patients in the clinic after wound infection, including defective bacteria clearance ability, more invasive infection, and more chronic wounds. Besides this, the overall situation (weight and blood glucose) of the diabetic rats obviously worsened after infection with *S. aureus*, while there were no differences in the normal rats. Furthermore, we found that *S. aureus* infection can trigger the autophagy of skin macrophages in both the normal and diabetic rats, however, the autophagic flux was blocked in the diabetic rats, which could play an important role in the bacterial internalization. Ongoing analysis of autophagy in the intracellular bacterium infection in diabetes will increase our knowledge about the inner immune response to the infection of DFU.

## 2 METHODS

### 2.1 Materials and bacterial strains

Monoclonal rabbit anti‐beta‐actin (ab8227), anti‐CD68 (ab125212), and monoclonal mouse anti‐LC3B (ab63817) were obtained from Abcam. Monoclonal mouse anti‐P62 (AF5384), goat antimouse immunoglobulin G (IgG) (H+L) horseradish peroxidase (HRP) (S0002), goat antirabbit IgG (H+L) HRP (S0001) were obtained from Affinity Biosciences. Cy3‐AffiniPure Goat antimouse IgG (H+L) (33208ES60), FITC‐AffiniPure Goat antimouse IgG (H+L) (33208EA33), and 4′,6‐diamidino‐2‐phenylindole (DAPI; 40727ES10) were obtained from Yeasen. *S. aureus* strain NCTC8325 was kindly provided by Dr. Wenjiao Chang (USTC). Bacteria were grown overnight at 37°C in Luria‐Bertani broth (Huankai). The optical density at 600 nm of bacterial cultures was measured to determine growth curves.

### 2.2 Animals and wounding

Animal experimental procedures were undertaken according to the protocols of the Guangdong Academy of Medical Sciences. Seventy‐two Sprague Dawley rats were allocated randomly into normal (*n* = 18), normal + infection (*n* = 18), diabetic (*n* = 18), or diabetic + infection (*n* = 18) groups by body weight. Diabetic groups were given streptozotocin (STZ; Sigma‐Aldrich; 65 mg/kg weight) injections and infection groups were inoculated with NCTC8325. After STZ injection, the blood glucose of tail vein was monitored with a blood glucose meter (Omron) once a week. STZ‐treated rats were considered to be diabetic when the concentration of blood glucose was equal to or higher than 16.7 mmol/L. The dorsal skin was shaved, treated with depilatory cream to remove hair, and then cleaned with a povidone‐iodine solution followed by an alcohol wipe. One circular, full‐thickness wound was created on the dorsal skin of each rat using a 10 mm biopsy punch. *S. aureus* NCTC8325 (5 × 10^8^ colony‐forming units [CFUs]) was applied to wounds of infected group animals, which were then covered with sterile gauzes and fixed with the sterile band. Rats were placed on a warming pad (37°C) until they fully recovered from surgery and were then re‐caged. On postoperative day (POD) 0, 4, 7, and 10 images of wounds with a ruler (six rats per time point per group) were collected by the camera (E‐M10; Olympus), and the wound area was analyzed by ImageJ. Wound healing rates were calculated according to the formula (wound area [POD 0] − wound area [POD N])/wound area (POD 0). Skin wound granulation tissue, liver, kidney, lung, and serum were isolated from rats as previously described. All the tissues were divided into three parts, then were frozen in liquid nitrogen or fixed in 4% formaldehyde, used to extract total protein or tissue stain respectively. White blood cell counts (WBC) were determined in whole blood. Finally, rats were anesthetized.

### 2.3 Bacterial burdens

At specific postinfection time points, bacteria were collected by rolling sterile swabs on the whole wound surface five times and then eluting in 1 ml of sterile phosphate‐buffered saline (PBS). Equal weights of wound tissue, the muscle under the wound, liver, kidney, and lung tissue were isolated and homogenized in 1 ml of sterile PBS. The swab elutions and tissue homogenates were then serially diluted in PBS and plated on tryptose soya agar (TSA) to determine the tissue bacterial burden.^18^, ^19^ Briefly, for swab culture, the PBS containing wound swab was eluted with vortex oscillation for 20 s × three times, and then continuously diluted with sterile PBS to make a series of diluted bacterial solutions of 10^−3^, 10^−4^, and 10^−5^. Within 20 min, 1 ml of eluent was added to a sterile TSA plate (three parallel plates were set for each concentration gradient). After 48 h culture at 37°C and 5% CO_2_, two operators used ImageJ (version 1.36b) to count the colonies on the TSA plate under the premise of double‐blind, and the results were taken as the average. For tissue culture, fresh tissue soaked in sterile PBS was dried with the sterile filter paper, then were weighed and recorded. The tissue was homogenized with a hand‐held electric homogenizer (Kimble), and then resuspended with 1 ml sterile PBS for homogenization, following by continuous dilution with sterile PBS in turn to make a series of diluted bacterial solutions of 10^−2^, 10^−3^, and 10^−4^. Within 20 min, 1 ml of tissue homogenate was incubated to the TSA and cultured as the swab culture. Populations of different bacterial species were differentiated by colony color, shape, and size and then confirmed by biochemical methods.

### 2.4 Hematoxylin and eosin staining of tissue

Tissue fixed by 4% formaldehyde was loaded by paraffin and sectioned with a thickness of 4 μm perpendicular to the head and tail axis and the skin surface, and baked at 60°C for 2 h to prevent paraffin flakes from falling off. After dewaxing by xylene, gradient alcohol dehydration (xylene I × 10 min, xylene II × 10 min, xylene III × 10 min, anhydrous alcohol I × 10 min, anhydrous alcohol II × 10 min, 95% alcohol × 5 min, 85% alcohol × 5 min, 75% alcohol × 5 min, double water rinsing, 5 min × 2 times), hematoxylin staining for 5–6 min, following by adequate washing, eosin staining for 2–3 min, washing with 70% alcohol until the color is moderate under the microscope. Go through radiant alcohol dehydration, xylene transparent, neutral resin after drying, finally cover the histological slides of tissue samples with slide seal, and observe with an optical microscope.

### 2.5 Western blot

Tissue samples were treated with lysis buffer contained phenylmethylsulfonyl fluoride after PBS washing. For Western blot, the tissue lysates were resolved by sodium dodecyl sulfate‐polyacrylamide gel electrophoresis and transferred to nitrocellulose membranes probed with anti‐LC3 (1:1000) and p62 (1:1000) monoclonal antibodies. After combination with the secondary antibodies (1:5000), the primary antibody's immunoreactive bands were detected by enhanced chemiluminescence, and signal values were normalized to the internal control (β‐actin).

### 2.6 Immunofluorescence staining

Immunofluorescence staining was performed to analyze the colocalization of LC3, p62, and CD68. Paraffin sections of skin tissue were stained with primary antibodies recognizing LC3 (1:1000), p62 (1:1000), or CD68 (1:500). After washing with PBS, the binding of the primary antibodies was revealed by Cy3‐AffiniPure Goat antimouse IgG (H+L) and FITC‐AffiniPure Goat antimouse IgG (H+L) and confocal laser scanning microscopy. CD68‐positive staining emitted bright green fluorescence, and LC3‐ or p62‐positive staining emitted red fluorescence. Nuclei were stained with DAPI. Controls for antibody specificity were included in all experiments; the primary antibody was replaced with PBS.

### 2.7 Statistical analyses

Results are presented as the mean ± standard deviation (*SD*). All experiments were repeated at least three times. Statistical analysis was performed with SPSS v20.0 (IBM). Student's *t*‐test and one‐way analysis of variance were used to analyze the differences in between‐group comparisons and multiple comparisons, especially. A two‐tailed *p* < .05 was considered significant.

## 3 RESULTS

### 3.1 **Wound infection by** *S. aureus* **obviously worsened the overall situation of the STZ rat skin wound model**

**Figure 1:**
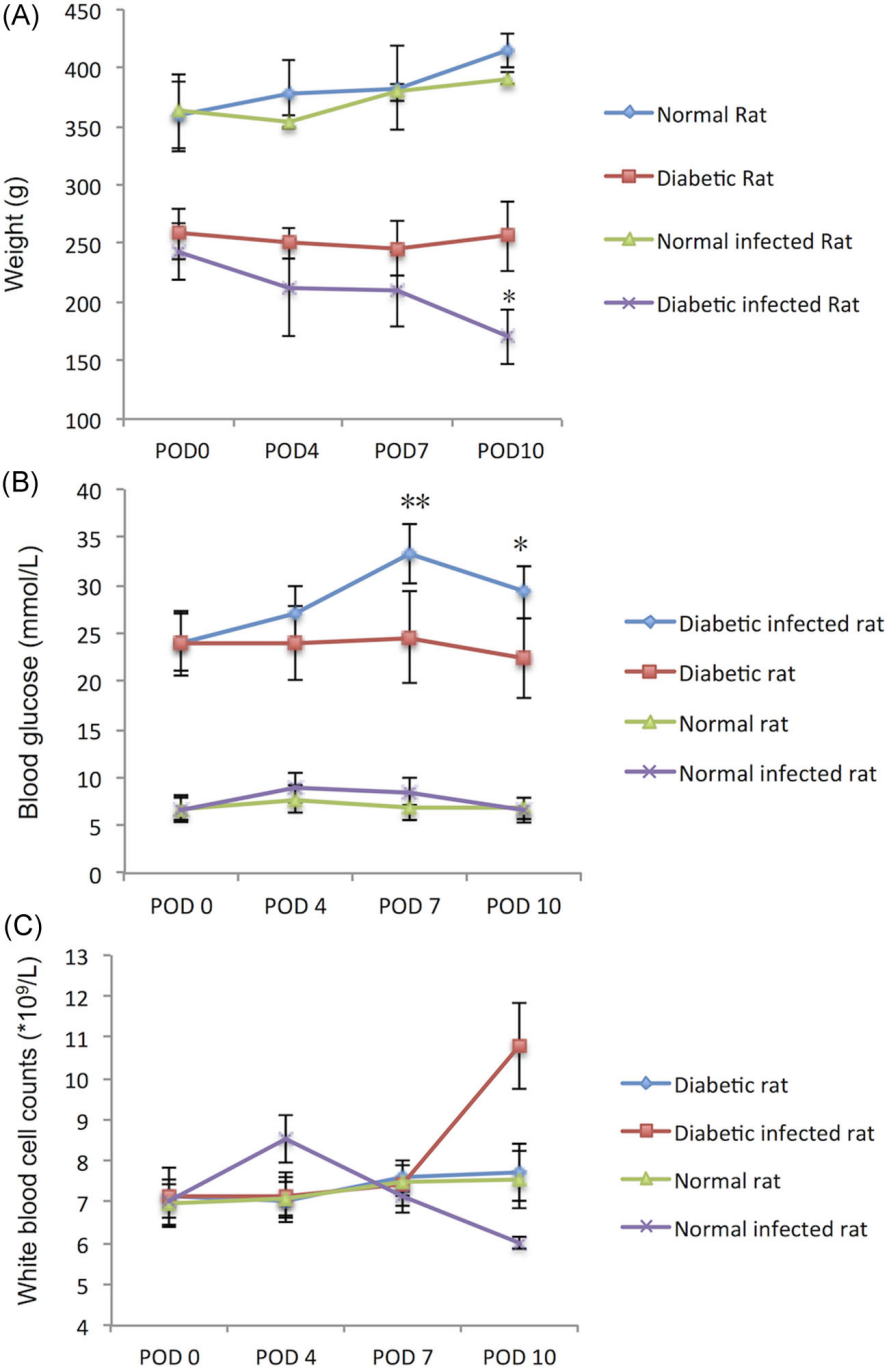
(A) Weight, (B) blood glucose, and (C) WBC of the rats. In diabetes rats, compared with diabetic rats on POD 0, *p < .05, **p < .01; in normal rats, compared with normal rats on POD 0, ^Δ^ *p* < .01. POD, postoperation day; WBC, white blood cell counts

Bodyweight and blood sugar were the main indexes to evaluate the overall situation of diabetic rats. As Figure 1A, B shows, there was no significant difference in both body weight and blood sugar in normal rats after wound infection with *S. aureus*. However, the blood sugar of diabetic rats increased significantly on POD 7 (29.27 ± 3.34 mmol/L for noninfection vs. 33.27 ± 2.78 mmol/L for infection; *p* < .01) and POD 10 (22.37 ± 3.34 mmol/L for noninfection vs. 24.57 ± 2.15 mmol/L for infection; *p* < .05) after infection. Compared with the noninfected group, bodyweight of infected diabetic rats was decreased significantly on POD 10 (170.33 ± 19.45 g for infection vs. 256.67 ± 26.21 g for noninfection; *p* < .05).

WBC in peripheral blood was an important index to evaluate the inflammation reaction to the infection. As Figure 1C shows, there was no significant difference (*p* > .05) between normal rats and diabetic rats in WBC when there was no infection. However, the WBC change trends appeared different between the two groups after *S. aureus* infection. For the normal rats, WBC showed a significant increase at the earlier stage (POD 4) ([8.54 ± 1.73] × 10^9^/L for infection vs. [7.11 ± 1.78] × 10^9^/L for noninfection; *p* < .05). But for the diabetic rats, the WBC peak appeared later, (10.78 ± 3.13) × 10^9^/L for infection versus (7.63 ± 2.09) × 10^9^/L for noninfection on POD10 (*p* < .05). Additionally, WBC of the rats with deep invasion and distant organ spread infection was significantly higher than that of the ones without spread ([9.84 ± 3.31] × 10^9^/L for infection versus [7.89 ± 2.47] ×  10^9^/L for noninfection; *p* < .05; Table S1).

### 3.2 *S. aureus* infection significantly delayed wound healing of the STZ rat skin wound model at the late‐stage

**Figure 2:**
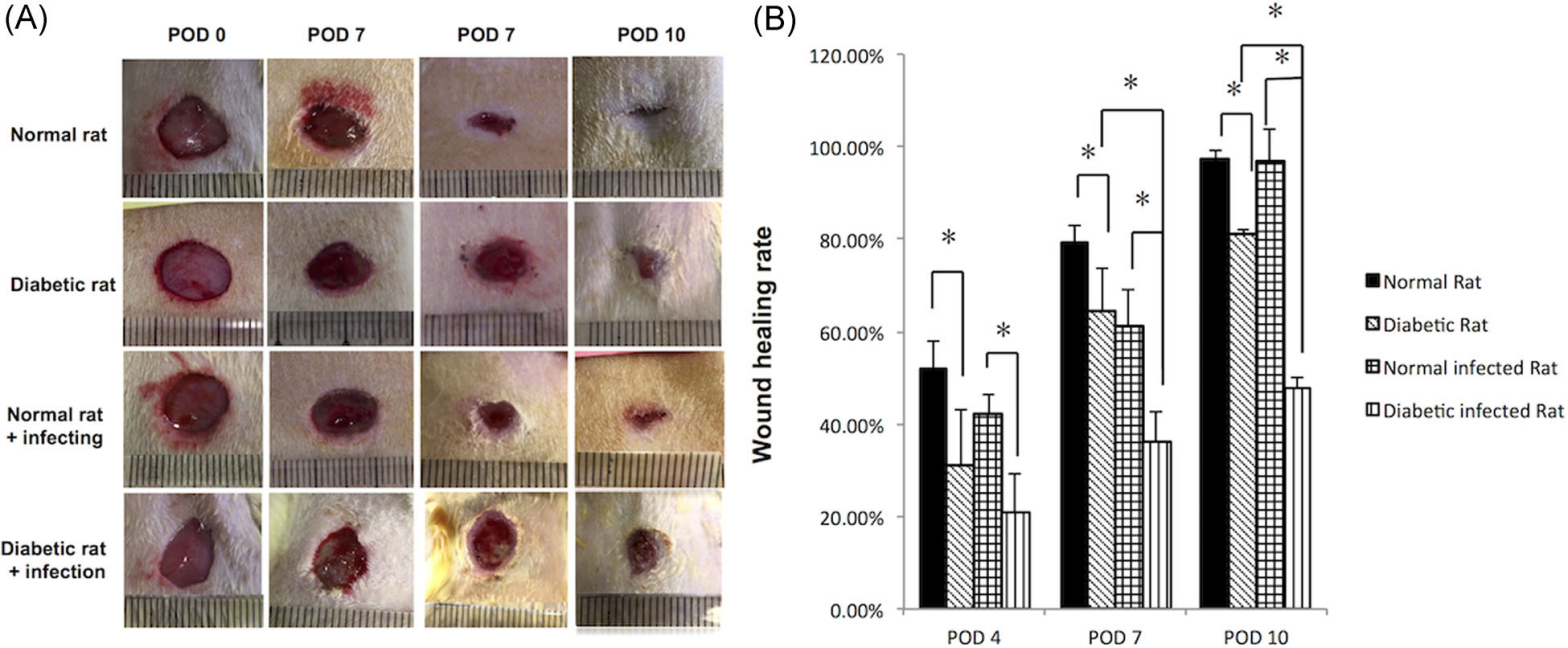
*Staphylococcus aureus* infection significantly delayed wound healing of the STZ rat skin wound model. (A) Wound images of normal rats and diabetic rats with and without infection at different postoperation days (PODs). (B) Comparison of wound healing rates (%) in different groups and different PODs. POD, postoperative day; STZ, streptozotocin. *p < .05, **p < .01

As per expectation, the skin wounds of diabetic rats were more difficult to heal compared with normal ones，with or without infection. After *S. aureus* infection, the normal rats' wound had slight purulent secretion on POD 4, but no obvious empyema after POD 7, with abundant granulation tissue and blood supply during the whole healing process. On the other hand, more obvious and lasting empyema and ischemic state could be seen on POD 4 and POD 7 in the diabetic rats' wound local after *S. aureus* infection (Figure 2A). *S. aureus* infection did not significantly affect the wound healing rate of normal rats (*p* > .05) but significantly delayed the wound healing of diabetic rats.

To the diabetic rats, there was no obvious difference in the wound healing rate between the infection group and the noninfection group at the early stage of wound infection (POD 4; *p* > .05); but at the late stages (POD 7 and POD 10), *S. aureus* infection significantly reduced the wound healing rates of diabetic rats, respectively 64.50 ± 9.00% for noninfection versus 36.00 ± 6.46% for infection (*p* < .05) on POD 7 and 81.20 ± 1.06% for noninfection versus 47.55 ± 2.41% for infection (*p* < .05) on POD 10. In normal rats, *S. aureus* infection had no significant impact on wound healing (*p* > .05). With infection, 83.33% (5/6) of the wound area was closed at the end of observation in the normal rats, while none (0/6) was closed in the diabetic rats (Figure 2B).

### 3.3 Bacterial killing ability was attenuated in the skin wounds of the STZ rat model

**Figure 3:**
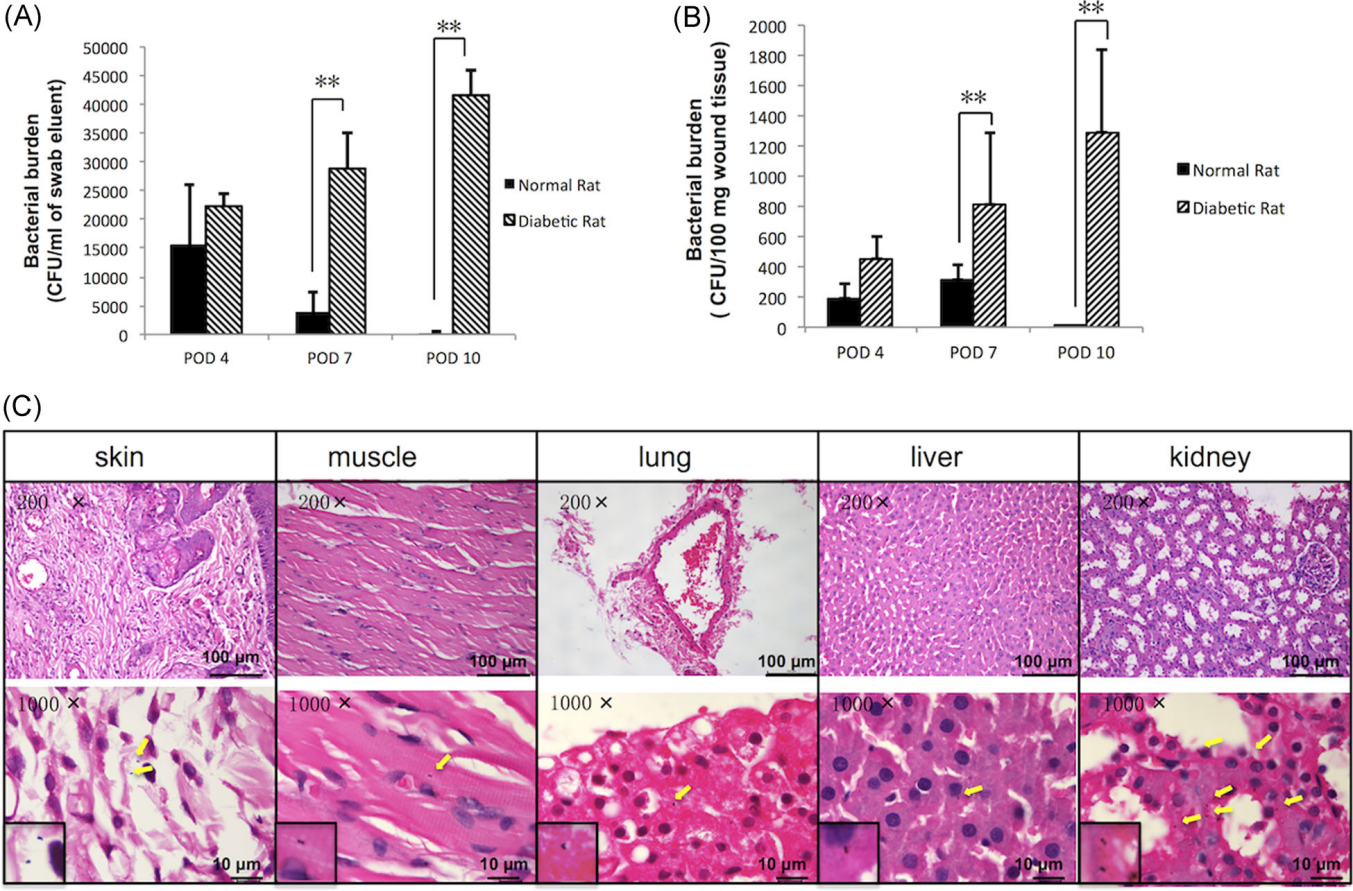
Bacterial killing ability is attenuated in STZ‐SD rat skin wounds. (A) The wound bacteria burden at POD 4, 7, 10 of normal and diabetic rats was assessed by swab culture and (B) tissue culture. *p < .05, **p < .01. (C) H&E staining of muscle under the wound, liver, kidney, and lung tissue of diabetic rats suffering from invasive infection or not. The yellow arrow indicates the presence of *Staphylococcus aureus* in the tissue. Data are expressed as the means with standard errors from three different experiments, and *t*‐tests were performed to validate statistical significance across conditions. CFU, colony‐forming unit; H&E, hematoxylin, and eosin; POD, postoperative day; STZ‐SD, streptozotocin‐induced Sprague Dawley. *p < .05, **p < .001

Throughout the process of the wound infection, higher wound surface bacterial burdens were observed in diabetic rats, together with elevated tissue bacterial load (Figure 3A,B).

The results of swab culture showed that the wound surface bacteria burden of diabetic rats on POD 7 was 73.54 times of the normal ones ([2.89 ± 0.06] × 10^5^ CFU/ml for infection vs. [3.93 ± 0.35] × 10^3^ CFU/ml for noninfection; *p* < .01). Further on POD 10, the increasing multiple rose to 138.66 times (*p* < .01). Wound tissue culture showed similar results. On POD 4, 7, and 10, the tissue bacteria burden of diabetic rats was significantly higher, respectively 2.35, 2.63, and 645.50 folds of the normal ones (*p* < .01). In addition, the changing trend of bacteria load in diabetic and normal rats appeared obviously different. The bacterial burden in the wound of normal rats decreased gradually with infection time, while the diabetic rats showed the opposite trend. The bacterial load of diabetic rats on POD 10 was 1.87 times (for wound surface) and 2.85 times (for wound tissue) higher than that on POD4, respectively.

Five deep muscle infections were found in the rats, four of which were diabetic rats (4/18) and one was normal (1/18). Five rats suffered from hematogenous spread infection to distant organs, all of which were diabetic rats, including two liver *S. aureus* infections (8.00 ± 0.67 CFU/100 mg tissue), five kidney *S. aureus* infections (8.33 ± 4.06 CFU/100 mg tissue) and two lung *S. aureus* infections (5.89 ± 5.52 CFU/100 mg tissue; Figure 3C and Table S1).

### 3.4 Impaired autophagy in the skin wounds of STZ rats

**Figure 4:**
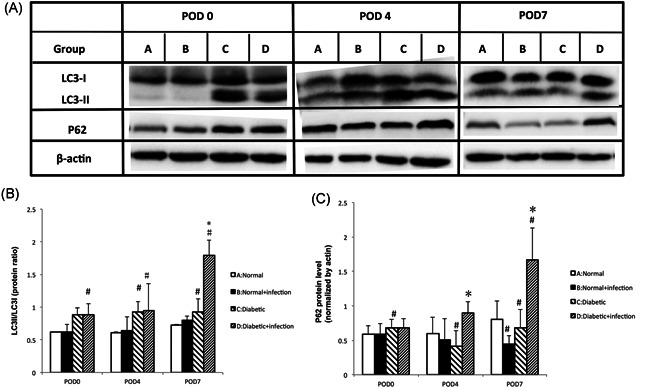
Impaired autophagy in STZ‐SD rat skin wounds. (A) Protein lysates of rat skin wounds were analyzed by Western blot analysis for LC3II/I and P62. Beta‐actin antibody was employed as a loading control. (B) Quantification of the conversion of LC3‐I to LC3‐II, and of P62. Data are expressed as the means with standard errors from three different experiments, and *t*‐tests were performed to validate statistical significance across conditions. POD, postoperative day; STZ‐SD, streptozotocin‐induced Sprague Dawley. Compared with the normal rats: ^#^ *p* < .05; Compared with the diabetic rats: *p < .05

As autophagy is the predominant way to clear intracellular pathogens, we examined LC3‐II and P62 expression to evaluate autophagy levels in the infected rat skin. In contrast to normal rats, the significant accumulation of LC3‐II and decreased P62 in wounded, noninfected diabetic rat skin on POD 4 and POD 7 indicated autophagy was triggered, and the autophagic flux was continuous (Figure 4).

*S. aureus* infection promoted autophagic flux in the skin tissues of normal rats on POD7, which featured increasing LC3‐II and further decreasing P62. Notably, in diabetic rats, effective autophagic flux was not induced by the *S. aureus* infection; in contrast, the significant accumulation of LC3‐II and P62 on POD4 and POD7 indicated the infection blocked autophagic flux in diabetic rat skin.

### 3.5 Autophagic flux of macrophages was blocked in STZ‐SD rat skin, especially after infection

**Figure 5:**
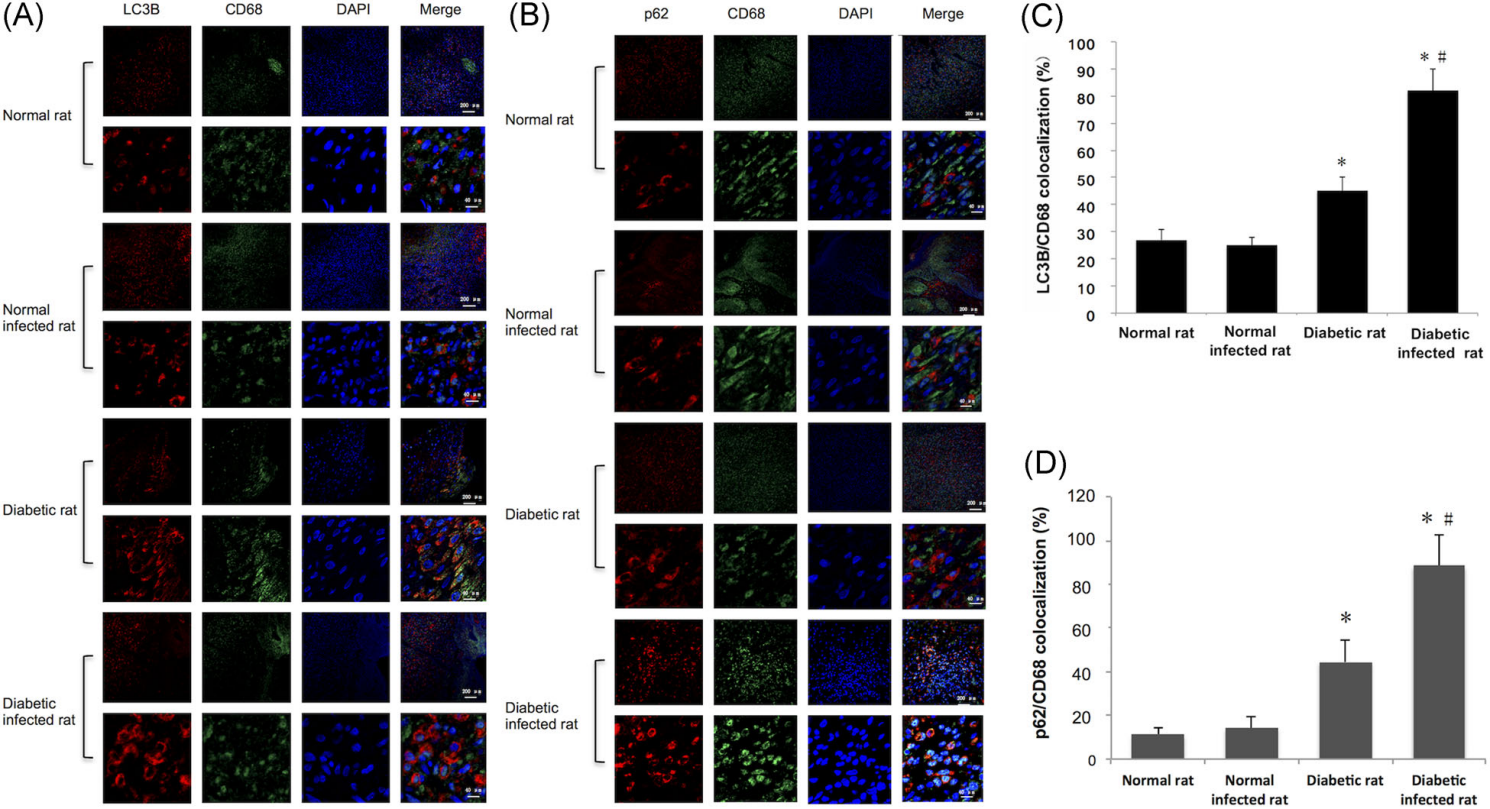
Autophagic flux of macrophages was blocked in STZ‐SD rat skin, especially after infection. (A) Immunofluorescence staining of LC3 (red fluorescence) and CD 68 (green fluorescence) of the wound skin of normal rat, normal infected rat, diabetic rat, and diabetic infected rat at POD 7, and the colocalization rate of LC3 and CD68 (C). (B) Immunofluorescence staining of P62 (red fluorescence) and CD68 (green fluorescence) of the skin of the four groups at POD 7, and the colocalization rate of P62 and CD68 (D). POD, postoperative day; STZ‐SD, streptozotocin‐induced Sprague Dawley. Compared with the normal rats: *p < .05; compared with the diabetic rats: ^#^ *p* < .05

To further investigate macrophages' autophagy levels in rat skin, immunofluorescence staining of LC3, P62, and CD68 was performed. The cytoplasmic localization of macrophage LC3 and P62 was obviously increased in diabetic rats compared with the normal group, which were more intensive in the infection groups, especially on POD 7 (Figure 5A, B). The colocalization rate of LC3‐II and CD68 in infected diabetic rats was 84.51 ± 4.22%, significantly higher than that in noninfected diabetic infected rats (44.32 ± 2.67%; *p* < .05), and meant that infection can trigger autophagy of dermis macrophages in diabetic rats (Figure 5C). But meanwhile, the colocalization rate of P62 and CD68 rose to 4.72‐fold after being infected by *S. aureus* (Figure 5D), suggested a blockage of autophagic flux.

## 4 DISCUSSION

DFI is a severe, common, and costly complication of diabetes, featured by wound healing and infection clearance disorders. As the most common Gram‐positive Cocci in DFI, *S. aureus* infection and biofilm formation^20^ are frequently found in diabetic chronic foot wounds with poor patients outcomes. However, the exact reasons for the prolonged infection of *S. aureus* of DFI are still unclear.^21^, ^22^ In the present study, we confirmed the unique infection performance when inoculated with the same dose of *S. aureus* in a rat skin wound model of diabetes.

Diabetic rats showed delayed wound healing, worsened overall situation, defective pathogen clearance, with increased invasiveness of the infections, which coincided with the DFI patients in the clinic.^23^ Many studies showed curettage (tissue scraping) with a dermal curette or scalpel from the base of a debrided ulcer, punch biopsy, or needle aspirate of purulent secretions, generally provided more accurate results than wound swabbing.^24^, ^25^, ^26^ To get more accurate and comprehensive data, we used both swab culture and wound tissue culture to evaluate the bacteria burden in this model. Results showed that in the premise of adequate wound debriding before sampling, swab culture could provide believable results in accordance with the tissue culture. So for the clinic, if swabs are the only available method, they should be taken only after debriding and cleaning the wound. In the clinic, osteomyelitis secondary to DFI is representative of invasive infection, whose predominant pathogen is *S. aureus*. Here, we confirmed that the diabetic rats were prone to suffer from invasive infection, manifested as the muscles infection, which was deep under the ulcer and subcutaneous fascia. Moreover, in this study, all the hematogenous disseminated infections (lung infection, kidney infection, and liver infection) secondary to skin infection occurred in diabetic rats, and the kidney is the most susceptible organ. These results indicated that the immune cell of diabetic rats could not kill or limit *S. aureus* effectively like the normal rats, causing infection to become more aggressive.

Besides this, some interesting change trends after infection were found in this model. To the normal rats, WBC in peripheral blood rose at a very early stage and reached the peak rapidly, which ensured fighting the pathogens in time. On the other hand, the bacteria burden in the wound decreased with the infection time, without antibacterial therapy, and the infection did not delay wound healing. Unfortunately, the diabetic rats showed a WBC increase at a very late stage, consequently the continuously increasing bacterial burden and significantly delayed healing. The results indicated that delayed inflammatory response and deficiency of professional immune cells could cause the impaired bacteria killing in DFI.

Macrophages are the predominant resident professional immune cells in the dermis,^27^ which play essential roles in the wound healing process, especially bacterial killing. Circulating peripheral‐blood mononuclear cells migrate into the wound area, and differentiate into macrophages to engulf and digest pathogens indiscriminately.^12^ The disturbance of the cutaneous environment, such as the high glucose and excessive AGEs accumulation in diabetic patients has been shown to alter the morphology, quantity, and function of the macrophages, all of which could delay wound healing.^28^

Wound healing is a complex process that depends on multiple factors, such as extracellular matrix, cytokines, inflammatory cells, and repair cells, highly coordinated in space and time. Autophagy affects wound healing over the whole procedure. In the early inflammatory phase, autophagy of the skin cells helps to eliminate pathogens in the wound by the xenophagy pathway, meanwhile, downregulates the inflammatory response to prevent tissue damage caused by excessive inflammation. In the following process of wound angiogenesis, autophagy can affect angiogenesis through multiple hypoxia signal pathways; however, excessive autophagy can lead to autophagic death of constituent cells in this procedure.^29^

Some researchers found that the autophagic flux in diabetic animal models were abnormal. Wang et al.^30^ observed increased autophagy in the retinas of type 1 diabetic rodents, and the activated autophagy promotes the death of ganglion cells, eventually leading to retinal degeneration in a rat model. Another team's work showed that the number of autophagosomes in the podocytes of diabetic nephropathy (DN) rats decreased obviously, which is involved in the pathogenesis of podocyte loss and leads to massive proteinuria in DN.^31^ In this study, we found the autophagic flux was obviously blocked in diabetic rats without wounds. However, the blocked autophagic flux was activated after the wound, furthermore, it presents an over‐activated state, compared with the normal rat wound. Excessive autophagy could lead to the lack of inflammatory response and chronic inflammation in the early stage of diabetic wounds. Moreover, it can cause a lack of repair cells due to autophagic death. After infection, the autophagy flux of skin macrophages in diabetic rats was obviously blocked; finally, the whole autophagy effect was inhibited. Consequentially, for lack of “negative regulation” of autophagy to the inflammatory response, the inflammation aggravates to cause tissue damage in the wound local. Additionally, the angiogenesis was affected as well due to the autophagy inhibition, delaying wound healing. More importantly, the blocking of autophagy flux of macrophages directly leads to weakening the killing ability of internalized pathogens.

The deficiency of host professional immune cells' intracellular bacteria‐killing ability promotes pathogen intracellularity, which plays a crucial role in the infection spreading and invasion. Selective autophagy of macrophages acts as an innate immune defense mechanism and a direct antimicrobial mechanism.^14^, ^32^, ^33^ Here, we found the autophagy in the dermal macrophages of STZ rats was impaired, especially after infection.

The autophagy process can be divided into three steps: autophagosomes formation, autolysosomes formation, and degradation. Briefly, when autophagy was triggered, the LC3‐II wound is raised to structure the biomembrane vesicles “autophagosomes” to enclose the pathogens first; second, after the autophagosomes fused with lysosomes to form autolysosome and finally the contents were degraded in the autolysosome, and meanwhile, p62 wound decrease. This whole process was called autophagic flux.^34^

In this study, we observed that autophagy activation already existed in diabetic rats' skin, especially with wounds, coincident with the previous study. The *S. aureus* infection further triggered the autophagy, but the p62 did not decline like that in an intact autophagic flux, instead of an obvious increase. That indicated some mistake existed in the fusion or degradation step. That would not only cause the *S. aureus* in the autophagosomes to avoid being killed but also be protected by this biomembrane vesicle to reproduce, invade, and disseminate. This can be explained by our published research results in vitro, which revealed that AGEs impaired the host macrophages' autophagic flux via elevating lysosomal Arl8 level in macrophage, a key negative regulator in perinuclear positioning of lysosomes when autophagosome‐lysosome fuse, finally inducing autophagosome formation but inhibiting the autolysosome formation.^17^

It was once believed that *S. aureus* was an extracellular infection, but recent research suggested that *S. aureus* is a facultative intracellular pathogen, which can survive in host cells that make the infection more invasive.^7^, ^35^ In previous studies, under certain conditions, specific *S. aureus* strains were shown to subvert host autophagy by triggering autophagosome formation while inhibiting the fusion of phagosomes with lysosomes, creating a protective niche for intracellular bacteria to grow and escape from the infected cells.^8^, ^9^ Compared with the “autophagy fight” approach of other intracellular pathogens, this special strategy has been named “autophagy hijacking.”^36^ This feature may differ by the strains of *S. aureus*, their growth phase at the time of infection, as well as MOI during the infection.^7^

In conclusion, our data showed that it is featured by delayed wound healing, defective pathogen clearance, increased invasiveness of the infections in the STZ rat skin wound infection model, and *S. aureus* impaired autophagic flux in the diabetic host macrophages by inducing autophagosome formation but blocking the subsequent course. The exact relationship and underlying mechanisms of impairment of autophagy and infection characteristics of DFI are still unclear.^37^ Further understanding of the special immune crosstalk between diabetes host and *S. aureus* infection through autophagic factors will help to explain the complex clinical phenomenon and guarantee the development of effective therapies for DFIs.

## AUTHOR CONTRIBUTIONS

Chaohui Duan, Li Yan, and Xiaoying Xie contributed to the design of the study and the writing of the manuscript. Rihui Zhong, Ling Luo, and Xianghua Lin performed animal experiments. Lisi Huang, Songyin Huang, Lijia Ni, Baiji Chen, and Rui Shen assisted with molecular experiments, image processing, and quantitative data collection. All authors read and approved the final version of the manuscript.

## CONFLICT OF INTERESTS

The author declares that there are no conflict of interests.

## ETHICS STATEMENT

Animal experimental procedures were undertaken according to the protocols of the Guangdong Academy of Medical Sciences.

## Supporting information

Supporting information.

## Data Availability

All data during the study appear in the submitted article.

## References

[1] Belefquih B , Frikh M , Benlahlou Y , et al. Diabetic foot infection in Morocco: microbiological profile. Wounds. 2016;28(3):89‐98. 26978862

[2] Jiang Y , Ran X , Jia L , et al. Epidemiology of type 2 diabetic foot problems and predictive factors for amputation in China. Int J Low Extrem Wounds. 2015;14(1):19‐27. 25573978 10.1177/1534734614564867

[3] Sangeorzan BJ . Residual infection after forefoot amputation in diabetic foot infection: is new information helpful even when negative? J Bone Joint Surg Am. 2018;100(17):1447. 30180051 10.2106/JBJS.18.00593

[4] Wukich DK , Hobizal KB , Brooks MM . Severity of diabetic foot infection and rate of limb salvage. Foot Ankle Int. 2013;34(3):351‐358. 23520292 10.1177/1071100712467980 PMC4016951

[5] Troisi N , Ercolini L , Chisci E , et al. Diabetic foot infection: preliminary results of a fast‐track program with early endovascular revascularization and local surgical treatment. Ann Vasc Surg. 2016;30:286‐291. 26370745 10.1016/j.avsg.2015.07.015

[6] Xie X , Bao Y , Ni L , et al. Bacterial profile and antibiotic resistance in patients with diabetic foot ulcer in Guangzhou, Southern China: focus on the differences among different Wagner's Grades, IDSA/IWGDF Grades, and ulcer types. Int J Endocrinol. 2017, 2017:8694903. 29075293 10.1155/2017/8694903 PMC5623783

[7] Fraunholz M , Sinha B . Intracellular *Staphylococcus aureus*: live‐in and let die. Front Cell Infect Microbiol. 2012;2:43. 22919634 10.3389/fcimb.2012.00043 PMC3417557

[8] Lopez de Armentia MM , Gauron MC , Colombo MI . *Staphylococcus aureus* alpha‐toxin induces the formation of dynamic tubules labeled with LC3 within host cells in a Rab7 and Rab1b‐dependent manner. Front Cell Infect Microbiol. 2017;7:431. 29046869 10.3389/fcimb.2017.00431 PMC5632962

[9] Liu PF , Cheng JS , Sy CL , et al. IsaB inhibits autophagic flux to promote host transmission of methicillin‐resistant *Staphylococcus aureus* . J Invest Dermatol. 2015;135(11):2714‐2722. 26134948 10.1038/jid.2015.254 PMC4641007

[10] Matejuk A . Skin immunity. Arch Immunol Ther Exp. 2018;66(1):45‐54. 10.1007/s00005-017-0477-3 PMC5767194 28623375

[11] Brancato SK , Albina JE . Wound macrophages as key regulators of repair: origin, phenotype, and function. Am J Pathol. 2011;178(1):19‐25. 21224038 10.1016/j.ajpath.2010.08.003 PMC3069845

[12] Weiss G , Schaible UE . Macrophage defense mechanisms against intracellular bacteria. Immunol Rev. 2015;264(1):182‐203. 25703560 10.1111/imr.12266 PMC4368383

[13] Khan A , Jagannath C . Analysis of host‐pathogen modulators of autophagy during *Mycobacterium tuberculosis* infection and therapeutic repercussions. Int Rev Immunol. 2017;36(5):271‐286. 28976784 10.1080/08830185.2017.1356924

[14] Palmer GE . Autophagy in the invading pathogen. Autophagy. 2007;3(3):251‐253. 17224622 10.4161/auto.3820

[15] Dagdas YF , Pandey P , Tumtas Y , et al. Host autophagy machinery is diverted to the pathogen interface to mediate focal defense responses against the Irish potato famine pathogen. eLife. 2018;7:e37476. 29932422 10.7554/eLife.37476 PMC6029844

[16] Bah A , Vergne I . Macrophage autophagy and bacterial infections. Front Immunol. 2017;8:1483. 29163544 10.3389/fimmu.2017.01483 PMC5681717

[17] Xie X , Yang C , Duan C , et al. Advanced glycation end products reduce macrophage‐mediated killing of *Staphylococcus aureus* by ARL8 upregulation and inhibition of autolysosome formation. Eur J Immunol. 2020;50(8):1174‐1186. 32250445 10.1002/eji.201948477

[18] Salehifar E , Khorasani G , Ala S . Time‐related concordance between swab and biopsy samples in the microbiological assessment of burn. Wounds. 2009;21(3):84‐88. 25903099

[19] Vural MK , Altoparlak U , Celebi D , Akcay MN . Comparison of surface swab and quantitative biopsy cultures dependent on isolated microorganisms from burn wounds. Eurasian J Med. 2013;45(1):34‐38. 25610245 10.5152/eajm.2013.05 PMC4261496

[20] Xie X , Liu X , Li Y , et al. Advanced glycation end products enhance biofilm formation by promoting extracellular DNA release through sigB upregulation in *Staphylococcus aureus* . Front Microbiol. 2020;11:1479. 32765439 10.3389/fmicb.2020.01479 PMC7381169

[21] Xie X , Bao Y , Ni L , et al. Bacterial profile and antibiotic resistance in patients with diabetic foot ulcer in Guangzhou, Southern China: focus on the differences among different Wagner's Grades, IDSA/IWGDF Grades, and ulcer types. Int J Endocrinol. 2017;2017(1):8694903. 29075293 10.1155/2017/8694903 PMC5623783

[22] Chen YE , Fischbach MA , Belkaid Y . Skin microbiota‐host interactions. Nature. 2018;553(7689):427‐436. 29364286 10.1038/nature25177 PMC6075667

[23] Noor S , Khan RU , Ahmad J . Understanding diabetic foot infection and its management. Diabetes Metab Syndr. 2017;11(2):149‐156. 27377687 10.1016/j.dsx.2016.06.023

[24] Lipsky BA , Pecoraro RE , Larson SA , Hanley ME , Ahroni JH . Outpatient management of uncomplicated lower‐extremity infections in diabetic patients. Arch Intern Med. 1990;150(4):790‐797. 2183732

[25] Nelson A , Wright‐Hughes A , Backhouse MR , et al. CODIFI (Concordance in Diabetic Foot Ulcer Infection): a cross‐sectional study of wound swab versus tissue sampling in infected diabetic foot ulcers in England. BMJ Open. 2018;8(1):e019437. 10.1136/bmjopen-2017-019437 PMC5879729 29391370

[26] Lipsky BA , Aragón‐Sánchez J , Diggle M , et al. IWGDF guidance on the diagnosis and management of foot infections in persons with diabetes. Diabetes Metab Res Rev. 2016;32(Suppl 1):45‐74. 26386266 10.1002/dmrr.2699

[27] Dupasquier M , Stoitzner P , van Oudenaren A , Romani N , Leenen PJ . Macrophages and dendritic cells constitute a major subpopulation of cells in the mouse dermis. J Invest Dermatol. 2004;123(5):876‐879. 15482474 10.1111/j.0022-202X.2004.23427.x

[28] Klessens CQF , Zandbergen M , Wolterbeek R , et al. Macrophages in diabetic nephropathy in patients with type 2 diabetes. Nephrol Dial Transplant. 2017;32(8):1322‐1329. 27416772 10.1093/ndt/gfw260

[29] Qiu P , Liu Y , Zhang J . Review: the role and mechanisms of macrophage autophagy in sepsis. Inflammation. 2019;42(1):6‐19. 30194660 10.1007/s10753-018-0890-8

[30] Wang W , Wang Q , Wan D , et al. Histone HIST1H1C/H1.2 regulates autophagy in the development of diabetic retinopathy. Autophagy. 2017;13(5):941‐954. 28409999 10.1080/15548627.2017.1293768 PMC5446066

[31] Wang X , Gao L , Lin H , et al. Mangiferin prevents diabetic nephropathy progression and protects podocyte function via autophagy in diabetic rat glomeruli. Eur J Pharmacol. 2018;824:170‐178. 29444469 10.1016/j.ejphar.2018.02.009

[32] Huang J , Brumell JH . Bacteria‐autophagy interplay: a battle for survival. Nat Rev Microbiol. 2014;12(2):101‐114. 24384599 10.1038/nrmicro3160 PMC7097477

[33] McEwan DG . Host‐pathogen interactions and subversion of autophagy. Essays Biochem. 2017;61(6):687‐697. 29233878 10.1042/EBC20170058 PMC5869863

[34] Randow F , Munz C . Autophagy in the regulation of pathogen replication and adaptive immunity. Trends Immunol. 2012;33(10):475‐487. 22796170 10.1016/j.it.2012.06.003 PMC3461100

[35] Armbruster NS , Richardson JR , Schreiner J , Klenk J , Gunter M , Autenrieth SE . *Staphylococcus aureus* PSM peptides induce tolerogenic dendritic cells upon treatment with ligands of extracellular and intracellular TLRs. Int J Med Microbiol. 2016;306(8):666‐674. 27616282 10.1016/j.ijmm.2016.09.002

[36] Lussignol M , Esclatine A. Herpesvirus and Autophagy: “All Right, Everybody Be Cool, This Is a Robbery!”. Viruses. 2017;9(12):372. 10.3390/v9120372 PMC5744147 29207540

[37] Guo Y , Lin C , Xu P , et al. AGEs induced autophagy impairs cutaneous wound healing via stimulating macrophage polarization to M1 in diabetes. Sci Rep. 2016;6:36416. 27805071 10.1038/srep36416 PMC5090347

