# Supplementary material for: The infection characteristics and autophagy defect of dermal macrophages in STZ‐induced diabetic rats skin wound Staphylococcus aureus infection model
Source: Immun Inflamm Dis. 2021 Oct 14;9(4):1428–38. doi: 10.1002/iid3.492 (PMC8589369; doi:10.1002/iid3.492)
Supplement: Supplementary file 1 — Supporting information. [file IID3-9-1428-s001.docx]

**Supplement Material:**

**Table S1.** Information of the rats infected by *S.aureus*.

Footnote: The rats suffered from invasive or distant disseminated infection were highlighted with gray background.
